# Update to the study protocol, including statistical analysis plan, for the multicentre, randomised controlled OuTSMART trial: a combined screening/treatment programme to prevent premature failure of renal transplants due to chronic rejection in patients with HLA antibodies

**DOI:** 10.1186/s13063-019-3602-2

**Published:** 2019-08-05

**Authors:** Dominic Stringer, Leanne M. Gardner, Janet L. Peacock, Irene Rebollo-Mesa, Rachel Hilton, Olivia Shaw, Richard Baker, Brendan Clark, Raj C. Thuraisingham, Matthew Buckland, Michael Picton, Judith Worthington, Richard Borrows, David Briggs, Sapna Shah, Kin Yee Shiu, Keith McCullough, Mysore Phanish, Janet Hegarty, John Stoves, Aimun Ahmed, Waqar Ayub, Robert Horne, Paul McCrone, Joanna Kelly, Caroline Murphy, Anthony Dorling

**Affiliations:** 1 Department of Biostatistics and Health Informatics, Institute of Psychiatry, Psychology and Neuroscience, King’s College London, De Crespigny Park, London, SE5 8AF UK; 2 MRC Centre for Transplantation, King’s College London, Guy’s Hospital, Great Maze Pond, London, SE1 9RT UK; 3 School of Population Health and Environmental Sciences, King’s College London, London, UK; 4 Department of Nephrology and Transplantation, Guy’s Hospital, Great Maze Pond, London, SE1 9RT UK; 5 Clinical Transplantation Laboratory, Viapath, Guys Hospital, Great Maze Pond, London, SE1 9RT UK; 6 Renal Unit, St James’s University Hospital, Beckett Street, Leeds, LS9 7TF UK; 7 Transplant Immunology, Level 09 Gledhow Wing, St James’s University Hospital, Beckett Street, Leeds, LS9 7TF UK; 8 Department of Renal Medicine and Transplantation, Barts Health NHS Trust, London, E1 1BB UK; 9 Clinical Transplantation Laboratory, The Royal London Hospital, 2nd Floor, Pathology and Pharmacy Building, 80 Newark Street, London, E1 1BB UK; 10 Department of Renal Medicine, Manchester Royal Infirmary, Oxford Road, Manchester, M13 9WL UK; 11 Transplantation Laboratory, Manchester Royal Infirmary, Oxford Road, Manchester, M13 9WL UK; 12 Renal Unit, University Hospital Birmingham, Edgbaston, Birmingham, B15 2LN UK; 13 NHSBT Birmingham, Vincent Drive, Edgbaston, Birmingham, B15 2SG UK; 14 King’s College London, London, SE5 9RJ UK; 15 UCL Department of Renal Medicine, Royal Free London NHS Foundation Trust, London, NW3 2QG UK; 16 York Teaching Hospital NHS Foundation Trust, York, YO31 8HE UK; 17 Renal Unit, Epsom and St Helier University Hospitals NHS Trust, Surrey, UK; 18 Renal Unit, Salford Royal NHS Foundation Trust, Salford, M6 8HD UK; 19 Renal Unit, Bradford Teaching Hospitals NHS Foundation Trust, Bradford, BD5 0NA UK; 20 Renal Unit, Lancashire Teaching Hospitals NHS Foundation Trust, Preston, PR2 9HT UK; 21 Renal Unit, University Hospitals Coventry and Warwickshire NHS Trust, Coventry, CV2 2DX UK; 22 Centre for Behavioural Medicine, UCL School of Pharmacy, University College London, London, WC1H 9JP UK; 23 King’s Health Economics, Institute of Psychiatry, Psychology & Neuroscience, King’s College London, De Crespigny Park, London, SE5 8AF UK; 24 King’s Clinical Trials Unit, King’s College London, London, UK

**Keywords:** Human leucocyte antigen antibodies, Renal transplantation, Randomised controlled trial, Graft failure, Immunosuppression, Time-to-event, Statistical analysis plan

## Abstract

**Background:**

Chronic rejection is the single biggest cause of premature kidney graft failure. HLA antibodies (Ab) are an established prognostic biomarker for premature graft failure so there is a need to test whether treatment decisions based on the presence of the biomarker can alter prognosis. The Optimised TacrolimuS and MMF for HLA Antibodies after Renal Transplantation (OuTSMART) trial combines two elements. Firstly, testing whether a routine screening programme for HLA Ab in all kidney transplant recipients is useful by comparing blinding versus unblinding of HLA Ab status. Secondly, for those found to be HLA Ab+, testing whether the introduction of a standard optimisation treatment protocol can reduce graft failure rates.

**Methods:**

OuTSMART is a prospective, open-labelled, randomised biomarker-based strategy (hybrid) trial, with two arms stratified by biomarker (HLA Ab) status. The primary outcome was amended from graft failure rates at 3 years to time to graft failure to increase power and require fewer participants to be recruited. Length of follow-up subsequently is variable, with all participants followed up for at least 43 months up to a maximum of 89 months. The primary outcome will be analysed using Cox regression adjusting for stratification factors. Analyses will be according to the intention-to-treat using all participants as randomised. Outcomes will be analysed comparing standard care versus biomarker-led care groups within the HLA Ab+ participants (including those who become HLA Ab+ through re-screening) as well as between HLA-Ab-unblinded and HLA-Ab-blinded groups using all participants.

**Discussion:**

Changes to the primary outcome permit recruitment of fewer participants to achieve the same statistical power. Pre-stating the statistical analysis plan guards against changes to the analysis methods at the point of analysis that might otherwise introduce bias through knowledge of the data. Any deviations from the analysis plan will be justified in the final report.

**Trial registration:**

ISRCTN registry, ID: ISRCTN46157828. Registered on 26 March 2013;

EudraCT 2012–004308-36. Registered on 10 December 2012.

## Update

This update relates to the OuTSMART trial protocol, a randomised controlled trial testing whether a combined, structured, biomarker-screening programme and optimised immunosuppression treatment regimen can reduce risk of graft failure in kidney transplant patients. This update should be read in conjunction with the original protocol publication [1].

### Summary of design

OuTSMART is a prospective, open-labelled, randomised, biomarker-based strategy (hybrid) trial design, with two arms stratified by biomarker (human leucocyte antigen (HLA) antibody (Ab)) status. Recruitment will take place in 12 renal transplant units, recruiting for 45 months with recruits followed up intensively for at least 32 months (maximum 64 months) and primary endpoint assessed by remote evaluation after 43 months post randomisation is achieved by all.

Recipients of cross-match-negative transplants aged 18–75 years, and longer than 1 year post transplant with an estimated glomerular filtration rate (eGFR) ≥ 30 will be recruited into the trial.

The first stratification will result from blood-test screening for HLA Ab. Approximately 35% will be HLA positive (Ab+), with ~ 65% HLA negative (Ab^−^). The HLA Ab+ patients will be further screened with single antigen beads to determine whether donor-specific antibodies (DSA) are present (~ 1/6 DSA and 5/6 non-DSA). Thus, biomarker stratification leads to three groups (DSA+, non-DSA+ and HLA Ab^−^).

The second stratification will be based on current immunosuppression to ensure balanced numbers already on tacrolimus (Tac) or mycophenolate mofetil (MMF) in each group. The final stratification will be by site.

**Fig. 1:**
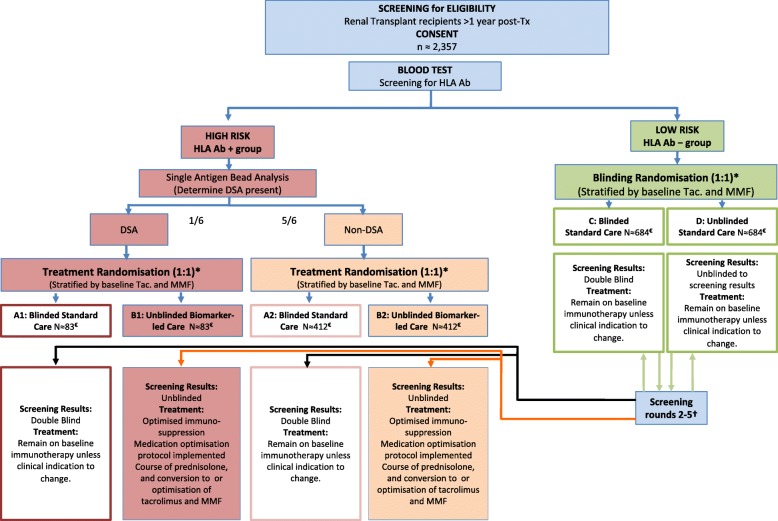
OuTSMART flow diagram. *Randomisation performed on results of a recruit’s first screening test. Those with human leucocyte antigen (HLA) antibodies (Ab) undergo no further screening as part of the trial (but serum will be stored for analysis of HLA Ab profiles later). †Those initially HLA Ab^−^ undergo routine screening every 8 months. There is no second randomisation: if a recruit allocated to blinded standard care (group C) becomes HLA Ab+ (black lines), they remain in the standard care group (group A1 or A2). If in unblinded standard care group (D), they change to unblinded biomarker-led treatment care (group B1 or B2) (orange lines). €Numbers in each group are those anticipated at the end of study

HLA Ab+ patients will be randomised 1:1 into either blinded standard care (SC) or unblinded biomarker led-care (BLC). Patients in the former (groups A1 and A2 in the flow chart in Fig. 1) will be blind to their biomarker status and will remain on baseline immunotherapy, whereas patients in the latter (groups B1 and B2 in Fig. 1) will know their HLA Ab status and will be offered ‘treatment’.

HLA Ab^−^ patients will remain on their existing immunotherapy and randomised 1:1 into either the blinded (group C) or the unblinded group (D), with only the latter knowing their HLA Ab status. Both these groups will receive regular Ab-status monitoring for the first 32 months. Those patients who become positive during subsequent screening rounds (~ 10% per year) will be moved to the appropriate HLA Ab+ groups (DSA+ or non-DSA+) for final data analysis (see Fig. 1).

All patients in group D found to be positive on second or subsequent rounds will be offered the same ‘treatment’ as those patients who were positive in the first screening round and be intensively followed up for an additional 32 months from the time that they become positive. Thus, the maximum amount of time that any single patient may remain in intensive follow-up is 64 months. New patients will be recruited to the study at each successive screening round.

The primary endpoint is time to graft failure in HLA Ab+ patients, where graft failure is defined as re-starting dialysis or requiring a new transplant.

### Change in primary outcome and sample size

The proportions of patients presenting with HLA Ab+ antibodies were closely monitored throughout recruitment to check that the assumptions and the sample size calculations were valid.

Sixteen months into recruitment (February 2015), prevalence (at randomisation) and incidence (through re-screening) rates of HLA Ab+ DSA were lower than originally expected. Nine percent prevalence and 3% incidence rates were expected [1] but 5.8% and 1.6% were observed, respectively. Consequently, it was estimated that approximately 4000 participants were needed to achieve the 278 HLA Ab+ DSA participants required for 80% power for the superiority hypothesis in HLA Ab+ DSA participants. This far exceeded our original estimated sample size of 2522 participants.

Therefore, we decided to change the primary outcome from graft failure rate at 3 years’ follow-up to time to graft failure. This allowed a reduction in the required sample size to 165 participants in the DSA group from an estimated 2357 participants overall. This reduction is achieved from the increased statistical power using time to graft failure which utilises more information from each observation [2].

These changes were approved by the OuTSMART Data Monitoring Committee (DMC), OuTSMART Trial Steering Committee (TSC), the London-Hampstead Research Ethics Committee (REC) and the Medicines and Healthcare products Regulatory Agency (MHRA).

### New sample size calculation

The updated sample size calculation was based on using time to graft failure as the primary endpoint and DSA prevalence and incidence rates observed from 16 months of recruitment. Other parameters were as per the original sample size calculation. The sample size calculation was carried out in Cytel’s East software v6.3 [3]

As previously, hypothesis testing will be sequential [1]. Superiority will be tested first in the HLA Ab+ groups to demonstrate the efficacy of the treatment. If this analysis is significant, then non-inferiority will be tested for the entire population to demonstrate the clinical utility of the biomarker screening. We calculated the sample size needed to show the superiority of the treatment optimisation for biomarker-positive patients using the hypotheses as follows:

*Hypothesis 1.1: HLA Ab+ patients with DSA, randomised to standard care (A1) will show higher graft failure rates than patients randomised to biomarker-led care (B1).*

We hypothesise that the experimental treatment will bring the failure rate of group B1 down to that of non-DSA patients in SC (A2). Assuming that a 30% failure rate for group A1 (as in [4]), and a 16% failure rate for group B1 at 3 years’ follow-up corresponds to a hazard ratio (HR) of 0.489, the expectation is for 11% and 21% chronic rejection (CR) rates among patients with DSA in group A1 at 1 and 2 years’ follow-up, respectively (as in [4]). Extrapolating, based on a HR of 0.489, we expect BLC to reduce those CR rates to 5.5% and 10.89% at 1 and 2 years, respectively. After 3 years’ follow-up, we assume a linear increase in the failure rate in group A1 per year (using the difference between the year-2 and year-3 failure rates; a 9% increase in the failure rate per year) with the same HR of 0.489.

Using a variable follow-up design, assuming an average accrual monthly rate of 3.6 patients per month and a minimum follow-up time of 43 months (with accrual period of 46 months for a maximum follow-up of 89 months), recruiting 165 patients with DSA would allow us to observe 23/83 (28%) events of CR in patients under BLC (B1), and 39/82 (47%) in the SC group (A1). This would provide 80% power and 5% type I error, for a two-sided log-rank test.

*Hypothesis 1.2: HLA Ab+ patients, with non-DSA, randomised to standard care (A2) will show higher graft failure rates than patients randomised to biomarker-led care (B2).*

We hypothesise that the experimental treatment will bring the failure rate of group B2 down to that of biomarker-negative patients in SC (C). Assuming a 16% failure rate for group A2 (as in [4]), and a 6% failure rate for group B2 at 3 years’ follow-up corresponds to a HR of 0.351. Based on Lachman et al. [4] the expectation is for 3% and 11% of CR among patients with non-DSA in SC at 1 and 2 years’ follow-up, respectively. Extrapolating based on a HR of 0.351, we expect BLC to reduce CR to 1.1% and 4.1% at 1 and 2 years, respectively. After 3 years’ follow-up, we assume a linear increase in the failure rate in group A2 per year (using the difference between the year-2 and year-3 failure rate; a 5% increase in the failure rate per year) with the same HR of 0.351.

Using a variable follow-up design (patients followed until failure, drop out or end of minimum follow-up), assuming an average accrual monthly rate of 15.5 patients per month, and a minimum follow-up time of 22.4 months, recruiting 296 patients with non-DSA, would allow us to observe 8/149 (5.3%) events of CR in patients under BLC, and 21/147 (14%) in the SC group (total duration = 41.5 months). This would provide 80% power to determine a statistically significant difference between SC and BLC, using a log-rank test, with a two-sided type-I error rate.

The numbers enrolled in groups A and B include those patients initially enrolled in groups C or D who become HLA Ab+ during re-screening.

*Hypothesis 2: All patients randomised to unblinded screening (combined groups B1 + B2 + D) will show equal or lower graft failure rates than all patients randomised to blinded screening (combined groups A1 + A2 + C), irrespective of biomarker status.*

We calculated the sample size needed to show the non-inferiority of all unblinded patients compared to all blinded patients as follows:

At the end of the trial, we expect 58% of patients to be in the HLA Ab^−^ groups, 7% DSA+ groups and 35% non-DSA+ groups (after drop outs). At the time of planning the OuTSMART study, we calculated that, based on these assumptions, all patients randomised to SC combined would experience 13.9% of CR. We established a non-inferiority limit of 5% absolute difference in rate of CR at 3 years, so that the BLC group would be considered inferior to SC with a CR rate of 18.9% or higher (expectation under the null hypothesis). This corresponds to a HR of 1.4 under the null hypothesis, and a HR of 0.63 under the alternative.

Recruiting 672 patients over a period of 13.2 months, at an average accrual rate of 51 patients per month, and a minimum follow-up of 18.21 months, would allow us to observe 22/337 (6.5%) events of CR in the SC group, and 32/335 (9.5%) in the BLC group. This would provide 90% power to demonstrate non-inferiority with a one-sided 95% confidence interval (CI) of the HR estimated using a Cox regression model. Given the above proportions, this requires enrolling 336 patients in each of groups C and D and this should allow 423 total HLA Ab^−^ patients to reach the primary endpoint.

#### Overall sample size

It was estimated that a total of 2357 participants were required to achieve the 165 HLA Ab+ DSA participants needed (using observed DSA incidence and prevalence rates). Because of this requirement to recruit sufficient DSA participants, the recruits to the other groups are likely to be more than the minimum required for statistical power for the individual hypotheses.

HLA Ab+ DSA rates continued to be monitored following these changes and observed rates increased. Recruitment was stopped once 165 HLA Ab+ DSA participants were reached (through randomisation or re-screening); 2037 participants were randomised in total. Re-screening continued until 8 months after the end of recruitment so that all participants were re-screened at least once.

### Change in follow-up

In the original protocol, participants were to be followed up every 4 months. This was reduced to 8-monthly visits to free up staffing resources for recruitment. Therefore, all data that was to be recorded every 4 months from randomisation will instead be recorded every 8 months. Each patient’s lipid profile will be measured every 16 months instead of every 12 months.

Length of follow-up was amended to be variable such that each participant will undergo 32 months of intensive follow-up post randomisation (or additionally 32 months after becoming HLA Ab+ at one of the re-screening rounds). Participants will be followed up with 8-monthly clinic visits in the intensive follow-up period. Subsequently, participants will be followed up remotely (with no additional contact other than routine care) until the last randomised participant reaches 43 months post randomisation As the accrual period is 46 months, participants will, therefore, be followed up for a maximum of 89 months.

In the 3 months prior to the conclusion of the study (the last randomised participant reaching 43 months post randomisation) all participants’ hospital records will be checked (other than participants already recorded as having graft failure, having died or having chosen to withdraw from data collection) for graft failure or death in the preceding follow-up period, with dates recorded if these events have occurred. If a participant does not have a routine clinic visit booked for this time period, the research staff at site will contact the participant by phone to determine whether graft failure has occurred. This assessment for each participant will only occur once in the given 3-month time period, at which point the participants’ involvement in the study will be completed.

### Change in secondary outcomes

With these changes, the list of secondary clinical endpoints has been amended to the following:

- Time to graft failure in patients randomised to blinded HLA Ab screening versus those randomised to unblinded screening. Graft failure will be defined as re-starting dialysis or requiring a new transplant
- Patient survival
- Graft dysfunction, as assessed by two separate measures, presence of proteinuria (protein/creatinine ratio (PCR) > 50 or albumin/creatinine ratio (ACR) > 35) and change in eGFRs over 32 months
- Rates of biopsy-proven rejection
- Rates of culture- or polymerase-chain-reaction-positive infection, biopsy-proven malignancy and diabetes mellitus
- Health economic analysis of outcomes
- Analysis of adherence and perceptions of risk for the BLC group

The definition of graft dysfunction as a secondary outcome has been amended. Originally, this was a composite of two binary endpoints defined as either the presence of proteinuria or a negative change in eGFR. Negative change in eGFR was a binary indicator defined as having a negative slope from a linear regression analysis of the serial eGFRs from baseline to 32 months and having to yield an adjusted R^2^ value of ≥ 0.35 and a *p* value of ≤ 0.05 [5]. Consequent to the reduction in number of study visits, there is a reduction in the number of eGFR measurements between baseline and 32 months. The reduction in the number of measurements would considerably reduce the probability of the linear regression being valid based on the criteria stated where there was a negative slope. This reduces the power to detect a difference (if one exists) between groups as there will be fewer events. A more powerful approach is to use a continuous measure of Modification of Diet in Renal Disease (MDRD) eGFR.

Proteinuria was defined as a PCR of > 50 from a urine sample. This definition has also changed as some sites use the ACR instead of the PCR.

Graft dysfunction will, therefore, be assessed by two separate measures, presence of proteinuria (PCR > 50 or ACR > 35) at 32 months and change in eGFRs over 32 months. eGFRs will be calculated using the MDRD. Mean eGFR slopes at 32 months will be compared between arms, using all available observations between baseline and 32 months in a linear mixed model.

Rates of acute rejection will still be assessed as stated in the original protocol except that rates will be assessed at the 32 months’ follow-up visit instead of at 3 years (36 months). There was no change to any of the other clinical outcomes.

The secondary scientific endpoints (change in HLA Ab characteristics, laboratory parameters of T- and B-cell phenotypes and responsiveness, numbers and phenotype of circulating CD34+) have been removed as formal secondary endpoints due to lack of resources.

### Change in inclusion/exclusion criteria

The inclusion criteria were amended to increase the age range from 18 to 70 to 18 to 75 years, on the realisation that many patients over 70 years of age are fit and healthy and thus could be considered for inclusion in the trial. ‘History of an ongoing or previous infection (no time limit) that would prevent optimisation of immunosuppression, including ocular *Herpes simplex*’ was removed as an exclusion criterion, after feedback that the vague nature of the criterion (for example, in not defining which infections were important) was being interpreted differently within and across sites and was, therefore, impacting on recruitment rates. As the optimisation for each participant was tailored to that individual, previous infections could be accommodated without excluding them. This was expected to standardise the selection of participants across all sites and increase recruitment rates.

## Statistical analysis plan

The statistical analysis plan has been revised in light of these changes and is outlined below.

All analyses will use the intention-to-treat population (all randomised participants included and according to allocated arm) unless otherwise stated.

Those patients who become positive during subsequent screening rounds will be moved to the appropriate HLA Ab+ groups (DSA+ or non-DSA+) for analysis. These participants will be included from the time that they became HLA Ab+ (date sample for screening was taken). For the analysis using all participants, these participants will be included from the time of randomisation.

This statistical analysis plan does not cover the health economic and health belief (adherence and perception of risk) outcomes which will not be reported in the primary paper but will instead be reported in separate papers.

### Data description

**Fig. 2:**
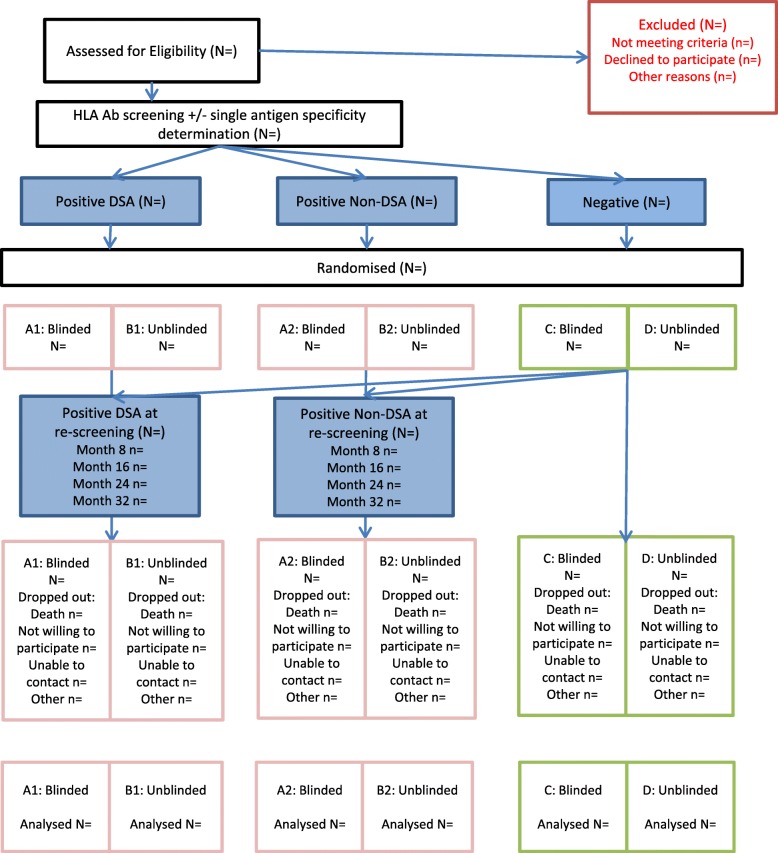
Consolidated Standards of Reporting Trials (CONSORT) diagram for OuTSMART. *Ab* antibody, *HLA* human leucocyte antigen, *DSA* donor-specific antibodies

A Consolidated Standards of Reporting Trials (CONSORT) flow chart will be constructed (Fig. 2). This will include the number of eligible patients, number of patients agreeing to enter the trial, number of patients refusing; number of HLA Ab+ and Ab^−^ patients, overall and by treatment arm: the number continuing through the trial, the number lost to follow-up or withdrawn, and the numbers excluded/analysed.

Baseline descriptions of participants will be tabulated by trial arm within HLA status and overall. Means and standard deviations (medians and inter-quartile ranges (IQR) if skewed) or numbers and proportions will be presented as appropriate. Especially relevant factors that will be tabulated are age, sex, HLA mismatches, baseline eGFR, site, previous treatment and time from transplantation. No significance testing will be used to test baseline differences between arms.

Frequencies and descriptive statistics of all primary and secondary outcome measures will be reported. Immunosuppressive drugs and doses prescribed to each group of patients will be summarised.

All descriptives will be broken down by HLA status. Where appropriate, descriptives for HLA Ab+ participants may be further broken down by whether they were HLA Ab+ at randomisation or through re-screening. Those who became HLA Ab+ at re-screening may also be described separately prior to becoming HLA Ab+ and after becoming HLA Ab+.

### Primary analysis

#### Superiority

- *H*_0_: *h*_*A1*_(*t*) = *h*_*B1*_(*t*) and *h*_*A2*_(*t*) = *h*_*B2*_(*t*)
- *H*_1_: *h*_*A1*_(*t*) ≠ *h*_*B1*_(*t*) and *h*_*A2*_(*t*) ≠ *h*_*B2*_(*t*)

To test superiority for the primary outcome in the biomarker (HLA Ab)-positive groups (hypotheses 1.1 and 1.2), we will use the Cox proportional hazards models to estimate the graft failure HR between the BLC and SC groups and test at the 5% level of significance. Results will be given as estimates and 95% CIs.

Within the model, we will adjust for previous immunosuppression regimen and research site (as these are the randomisation stratification factors) for increased statistical efficiency. We will check the proportional hazards assumption by examining Kaplan-Meier plots and by testing for an interaction between group (BLC or SC) and time to graft failure within the models.

#### Non-inferiority

- *H*_0_: *h*_Unblind_(*t*)/*h*_Blind_(t) ≥ *δ*
- *H*_1_: *h*_Unblind_(*t*)/*h*_Blind_(*t*) < *δ*

To test for non-inferiority of the unblinded groups compared to the blinded groups (hypothesis 2.1), we will use a Cox proportional hazards model to estimate the graft failure HR. We will adjust for the stratification factors in the model as outlined above and check the proportional hazards assumption by examining Kaplan-Meier plots and by testing for an interaction between group (unblinded/blinded) and time to graft failure. We will conclude non-inferiority if *H*_0_ gets rejected at 5% significance, and the corresponding upper bound of the 95% CI for the HR excludes the limit *δ* (HR of 1.4).

### Secondary analyses

We will use a similar procedure using Cox regression for the analysis of secondary time-to-event (survival) outcomes. Where numbers allow, secondary binary outcomes will be analysed using logistic regression with adjustment for stratification factors. Where numbers are too small for this, a Z-test or Fisher’s exact test will be used. For continuous secondary outcomes, linear regression will be used (or linear mixed models where accounting for repeated measures), adjusting for baseline values of the outcome and stratification factors. Transformations will be considered where data is skew.

Results will be given as estimates (odds ratios or differences in proportions) and 95% CIs.

No formal adjustment of *p* values for multiple testing is necessary. However, care will be given to the interpretation of inference for the numerous secondary outcomes.

### Harms

Adverse events (AE), adverse reactions (AR), serious adverse events (SAE) and serious adverse reactions (SAR) will be summarised by group (within trial arm and HLA status) as proportions and 95% CIs, broken down by body system code where there are sufficient numbers.

### Missingness

Missing baseline data should not be an issue for the primary analysis. Some of the secondary or exploratory analyses may use other baseline variables; if these contain missing data, the number with complete data will be reported and they will be imputed using a method suitable to the variable as per the recommendations of White and Thompson [6].

For missing outcome data, if post-treatment variables, such as compliance with treatment, are found to be predictive of drop out, multiple imputation will be considered.

### Per-protocol analyses

An exploratory per-protocol analysis will be carried out comparing time to graft failure in only those participants who were optimised to the full treatment protocol (as defined in [1]) in the unblinded arm against all blinded participants, within both the HLA Ab+ DSA and HLA Ab+ non-DSA groups.

In addition, if there are concerns about compliance, complier average causal effect (CACE) analysis [7] will be considered as a less biased way of estimating treatment efficacy.

### Exploratory/subgroup analyses

The trial protocol states that where, because of insufficient data on donor mismatches, for instance, the laboratory has difficulty labelling an HLA Ab as either a DSA or non-DSA, the Ab will be regarded as a non-DSA. Therefore, it is likely that the non-DSA group will contain patients with actual DSA. Therefore, an exploratory subgroup analysis will be carried out within the HLA Ab+ non-DSA group using only those participants within this group that have definite non-DSA.

Exploratory analyses will clearly be stated as such in any output and will be interpreted accordingly.

There are no other planned subgroup analyses.

### Interim analyses

There are no planned interim analyses.

### Software

Data management: an online data collection system for clinical trials (MACRO; Elsevier) will be used. This is hosted on a dedicated server at King’s College London (KCL) and managed by the Kings Clinical Trials Unit (KCTU).

Statistical analysis: Stata version 15.1 [8] and/or R [9] will be used for all statistical analyses.

## Discussion

This manuscript was written based on OuTSMART protocol version 13 and Statistical Analysis Plan version 2.2. This statistical analysis plan publication will help to avoid bias; pre-stating that the analysis plan helps to prevent changes to the analysis methods arising from knowledge of the data that may introduce bias. If there are any deviations from this statistical analysis plan, these will be outlined and justified in the final report.

### Trial status

OuTSMART has now completed recruitment and participants are in follow-up until June 2020. All HLA re-screening has also been completed. Two thousand and thirty-seven participants were randomised, of which 198 are HLA Ab+ with DSA following re-screening.

## Data Availability

Not applicable.

## Abbreviations

Ab: Antibodies
ACR: Albumin/creatinine ratio
BLC: Biomarker-led care
CR: Chronic rejection
DSA: Donor-specific antibodies
eGFR: Estimated glomerular filtration rate
EME: Efficacy and mechanism evaluation
HLA: Human leucocyte antigen
KCTU: King’s Clinical Trials Unit
MDRD: Modification of Diet in Renal Disease
MHRA: Medicines and Healthcare Products Regulatory Agency
MMF: Mycophenolate mofetil
NHS: National Health Service
NIHR: National Institute for Health Research
PCR: Protein/creatinine ratio
SC: Standard care
Tac: Tacrolimus
